# The small molecule drug diminazene aceturate inhibits liver injury and biliary fibrosis in mice

**DOI:** 10.1038/s41598-018-28490-y

**Published:** 2018-07-05

**Authors:** Indu G. Rajapaksha, Kai Y. Mak, Ping Huang, Louise M. Burrell, Peter W. Angus, Chandana B. Herath

**Affiliations:** 1 Department of Medicine, The University of Melbourne, Austin Health, Heidelberg, Victoria Australia; 2 Department of Gastroenterology, Austin Health, Heidelberg, Victoria Australia

## Abstract

There is no established medical therapy to treat biliary fibrosis resulting from chronic inflammation in the biliary tree. We have recently shown that liver-specific over-expression of angiotensin converting enzyme 2 (ACE2) of the renin angiotensin system (RAS) ameliorated liver fibrosis in mice. Diminazene aceturate (DIZE), a small molecule drug approved by the US Food and Drug Administration, which is used to treat human trypanosomiasis, has been shown to have antifibrotic properties by enhancing ACE2 activity. In this study we sought to determine the therapeutic potential of DIZE in biliary fibrosis using bile duct ligated and multiple drug resistant gene-2 knockout mice. Additionally, human hepatic stellate (LX-2) and mouse Kupffer (KUP5) cell lines were used to delineate intracellular pathways. DIZE treatment, both *in vivo* and *in vitro*, markedly inhibited the activation of fibroblastic stellate cells which was associated with a reduced activation of Kupffer cells. Moreover, DIZE-inhibited NOX enzyme assembly and ROS generation, activation of profibrotic transcription factors including p38, Erk1/2 and Smad2/3 proteins and proinflammatory and profibrotic cytokine release. These changes led to a major reduction in biliary fibrosis in both models without affecting liver ACE2 activity. We conclude that DIZE has a potential to treat biliary fibrosis.

## Introduction

Chronic cholestatic liver diseases and their sequelae of tissue fibrosis, cirrhosis, liver failure and portal hypertension remain a leading cause of chronic illness and death^1^. There are no established medical therapies for most of these diseases except for liver transplantation^2,3^. As a result, there is a major need to develop antifibrotic therapies which can prevent liver scarring and the development of cirrhosis in patients with these conditions.

Diminazene aceturate (DIZE; 4-[2-(4-carbamimidoylphenyl) iminohy-drazinyl]benzenecarboximidamide) is a US Food and Drug Administration (FDA)-listed small molecule which has been successfully used as a veterinary drug to treat diseases caused by blood-transmitted protozoan parasites such as Trypanosoma and Babesia species since it was first introduced more than six decades ago^4^. This drug is also used to treat human trypanosomiasis and appears to have no major toxicity^4–6^. Recently it has been proposed that DIZE activates ACE2, likely resulting in increased angiotensin-(1-7) (Ang-(1-7)) generation from angiotensin II (Ang II)^7^, which produces beneficial effects in a number of animal models including experimental pulmonary hypertension, myocardial infarction, and kidney disease^5,8–11^. Despite evidence to suggest DIZE is an ACE2 activator, a study by Haber and colleagues has shown that the beneficial effects elicited by the drug could be ACE2-independent^12^. This was based on the observations that DIZE and another putative ACE2 activator drug, 1-[[2-(dimetilamino)ethyl]amino]-4-(hidroximetil)-7-[[(4-metilfenil)sulfonil]oxi]-9H-xantona-9 (XNT), failed to enhance Ang II degradation by recombinant ACE2 both *in vitro* and *ex vivo*. Moreover, XNT reduced blood pressure in Ang II-induced acute hypertension in mice without affecting plasma or kidney ACE2 activity and produced the same beneficial effects in ACE2-knockout mice^12^.

The effects of DIZE on liver fibrosis and the renin angiotensin system have not been studied in liver disease. We therefore investigated if this potential therapeutic agent inhibits liver injury and fibrosis in cholestatic liver disease and whether it exerts effect via ACE2 activation. Here we used two different biliary fibrosis models; the multiple drug resistant gene-2 knockout (Mdr2-KO) mouse model which spontaneously develops severe and progressive biliary fibrosis due to defective bile acid secretion and the 2 weeks bile duct ligation (BDL) model. We show that DIZE is a potent antifibrotic agent in biliary disease and its effects are independent of ACE2 activation.

## Results

### DIZE reduces liver injury and inhibits biliary fibrosis in short- and long-term models

Both the BDL and the Mdr2-KO mice showed significantly elevated plasma levels of ALT, AST and ALP (Table 1) compared with those in sham-operated healthy controls or healthy FVB/N mice. However, in both models of biliary fibrosis, DIZE treatment caused a significant reduction in ALT, AST and ALP compared to saline treated BDL and Mdr2-KO control mice (Table 1), suggesting that DIZE improves liver injury.

**Table 1:** Liver biochemistry in diminazene aceturate (DIZE) treated mice with biliary fibrosis.

Animal group	Liver enzyme		
	ALT (U/L)	AST (U/L)	ALP (U/L)
Sham	12.58 ± 4.19	81.39 ± 22.57	46.85 ± 12.99
BDL + Saline	92.38 ± 32.66*	440.4 ± 139.27*	343.48 ± 91.8*
BDL + DIZE	57.11 ± 19.04*^,#^	307.2 ± 97.15*^,#^	254.7 ± 80.54*^,#^
Healthy control	18 ± 4.65	94.53 ± 24.41	30.13 ± 7.78
Mdr2-KO + Saline	152.47 ± 39.37*	405.47 ± 104.69*	231.8 ± 59.85*
Mdr2-KO + DIZE	79.71 ± 21.3*^,#^	250.14 ± 66.85*^,#^	149.83 ± 43.25*^,#^

Notes: *p < 0.05, significantly higher than respective sham or healthy control, ^#^p < 0.05, significantly lower than respective saline infused mice.

**Figure 1:**
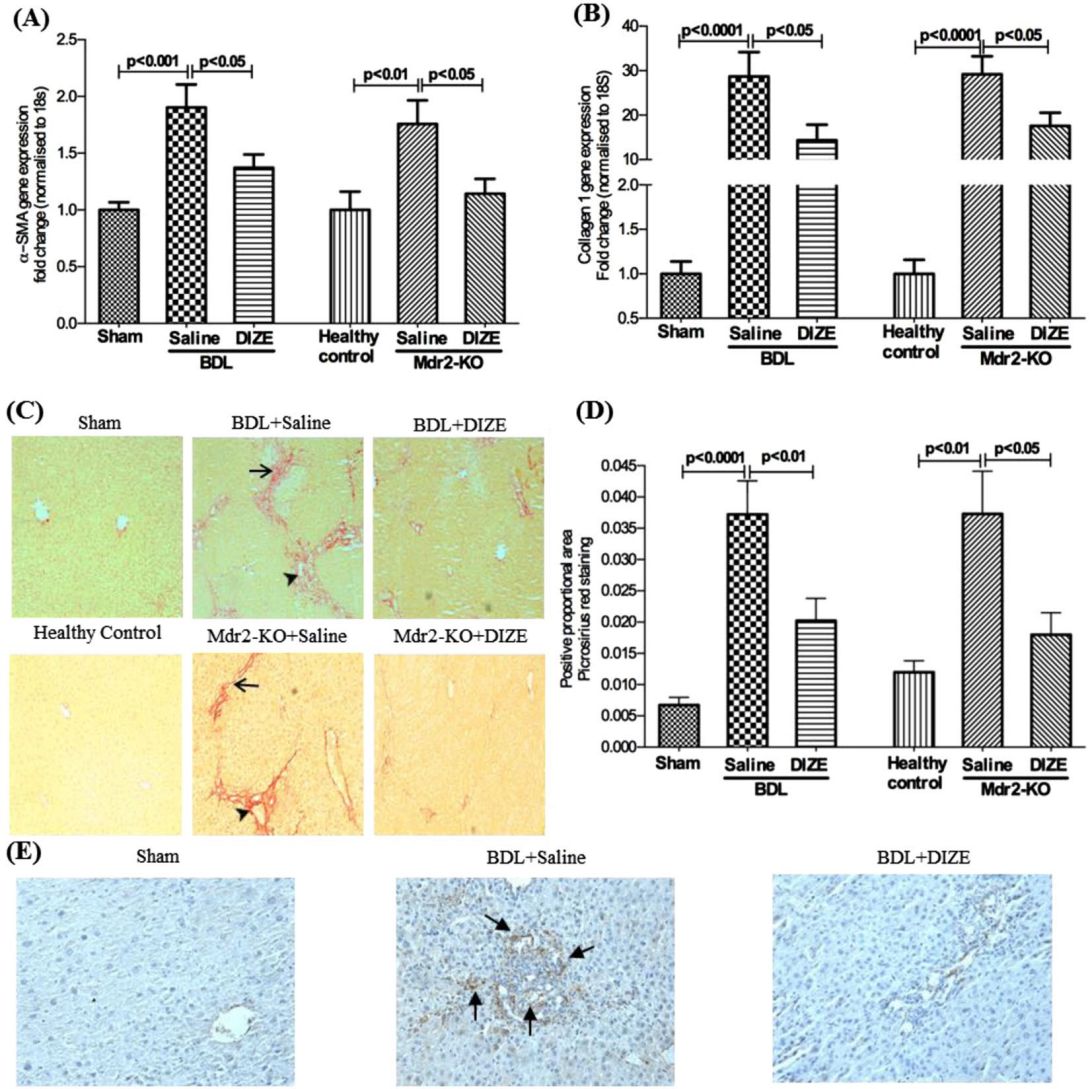
Diminazene aceturate (DIZE) treatment inhibited hepatobiliary fibrosis in mice. DIZE reduced the activation of hepatic stellate cells, as reflected by a reduced expression of α-smooth muscle actin (α-SMA) (**A**,**E**) and extracellular matrix protein, collagen 1 (**B**) leading to a marked reduction in biliary fibrosis (**C**,**D**) in both BDL and Mdr2-KO mice. Positive brown color staining for liver α-SMA by immunohistochemistry (arrows in panel E) and picrosirius red staining of bridging fibrosis (arrows in panel C) and fibrosis around portal triads (arrow heads in panel C) are shown (×200). Each bar represents the mean ± SEM profile from 10–15 mice per treatment group.

We assessed the presence of activated myofibroblasts by quantifying α-SMA. We found that α-SMA gene expression was significantly (p < 0.01) higher in BDL and Mdr2-KO mice than respective controls and that DIZE treatment caused a significant down-regulation of its expression (Fig. 1A). In accordance with the gene expression data, immunohistochemical localization of α-SMA protein in BDL livers showed that DIZE markedly reduced protein staining compared to that in saline infused animals (Fig. 1E).

We then assessed the antifibrotic effects of DIZE in these models by quantifying hepatic collagen 1 (type 1α1) gene expression by qPCR and protein deposition by picrosirius red staining. As expected, BDL caused a significant increase in collagen 1 gene expression (Fig. 1B) and collagen deposition (Fig. 1C,D) in the liver compared to sham-operated mice. Similarly, Mdr2-KO mice also showed significantly elevated collagen gene expression (p < 0.0001) (Fig. 1B) and collagen deposition (Fig. 1C,D) compared to FVB/N healthy controls. However, treatment with DIZE inhibited collagen 1 gene expression in BDL and Mdr2-KO mice (Fig. 1B). Consistent with reduced collagen 1 gene expression, DIZE markedly reduced collagen deposition as assessed by picrosirius staining in the diseased livers compared to untreated BDL and Mdr2-KO livers (Fig. 1C,D).

### DIZE inhibits tumour necrosis factor-α (TNF-α) expression and hepatocyte necrosis in BDL mice

**Figure 2:**
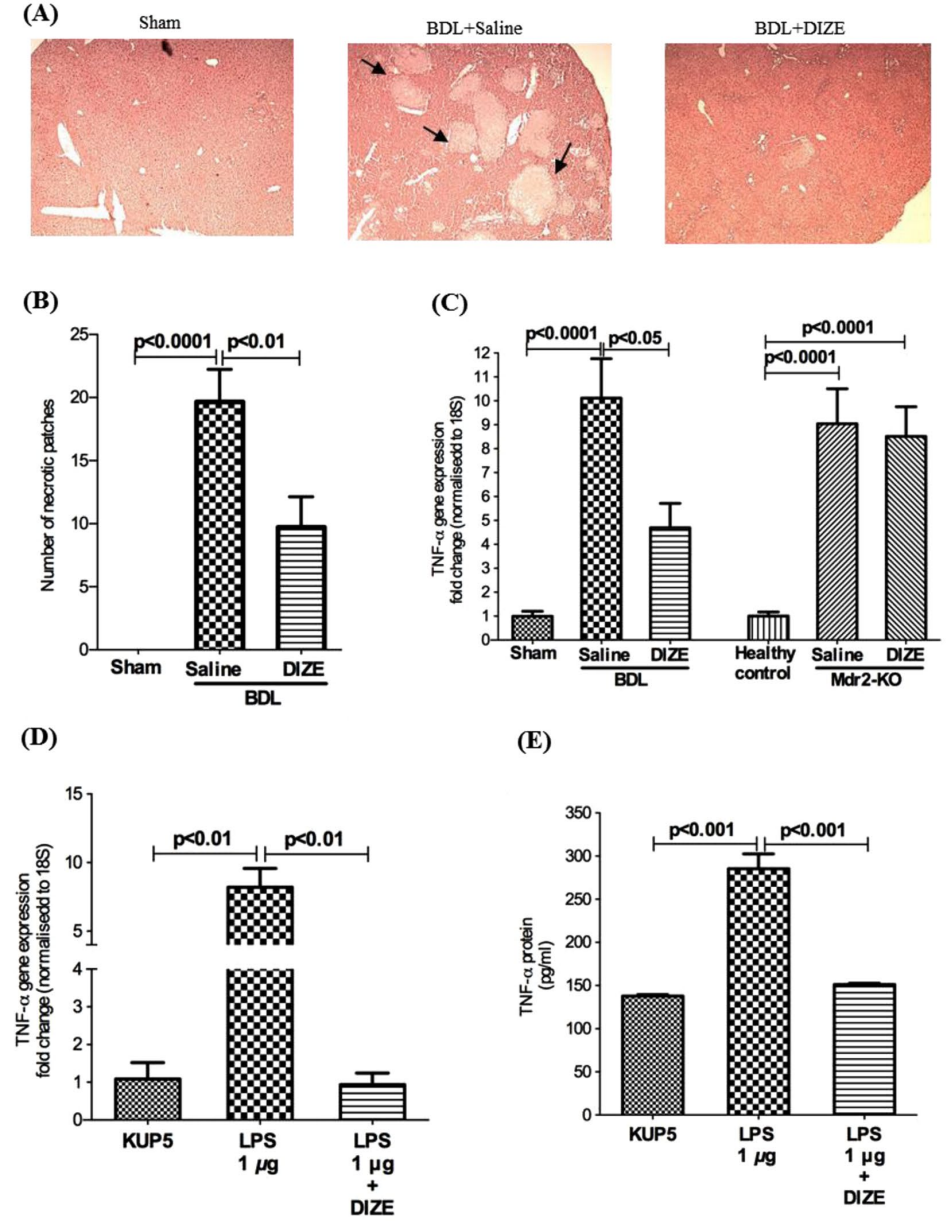
Diminazene aceturate (DIZE) treatment inhibited hepatocyte necrosis in BDL mice. H&E stained liver sections (**A**) (×40) showed more than 50% reduction in hepatocyte necrosis in DIZE treated BDL mice compared to saline treated BDL mice. (**B**) This reduction was associated in BDL but not in Mdr2-KO mice with a reduced expression of hepatic TNF-α. (**C**) Treatment with DIZE inhibited both gene expression (**D**) and protein secretion (**E**) of TNF-α by lipopolysaccharide (LPS)-stimulated Kupffer cells (KUP5 cells). Each bar represents the mean ± SEM profile from 10–15 mice (panel B,C) or 3 independent experiments (panels D,E) per treatment group. Arrows in panel A indicate necrotic patches in BDL mice.

As has been found in other studies in the BDL model^13,14^, we found that there were extensive necrotic areas in BDL but not in Mdr2-KO livers (Fig. 2A). However, DIZE treatment reduced the number of these areas by more than 50% (p < 0.01) (Fig. 2B). Since TNF-α is closely linked to hepatocyte necrosis, we investigated TNF-α expression in the liver. The expression of this death ligand was increased in BDL and Mdr2-KO mice compared to that in sham-operated or healthy controls; however, DIZE inhibited its expression in BDL mice (Fig. 2C) in parallel with its effects on hepatocyte necrosis. Because activated Kupffer cells are a major source of TNF-α in the liver, we investigated the secretion of this death ligand in KUP5 cells. We found that LPS caused an 8-fold increase in TNF-α gene expression (Fig. 2D) and 2-fold increase in its protein secretion (Fig. 2E). Confirming its effect in the liver, DIZE completely abrogated both gene expression and protein secretion of TNF-α by KUP5 cells (Fig. 2D,E).

### DIZE ameliorates the expression of pro-fibrotic and proinflammatory cytokines

**Figure 3:**
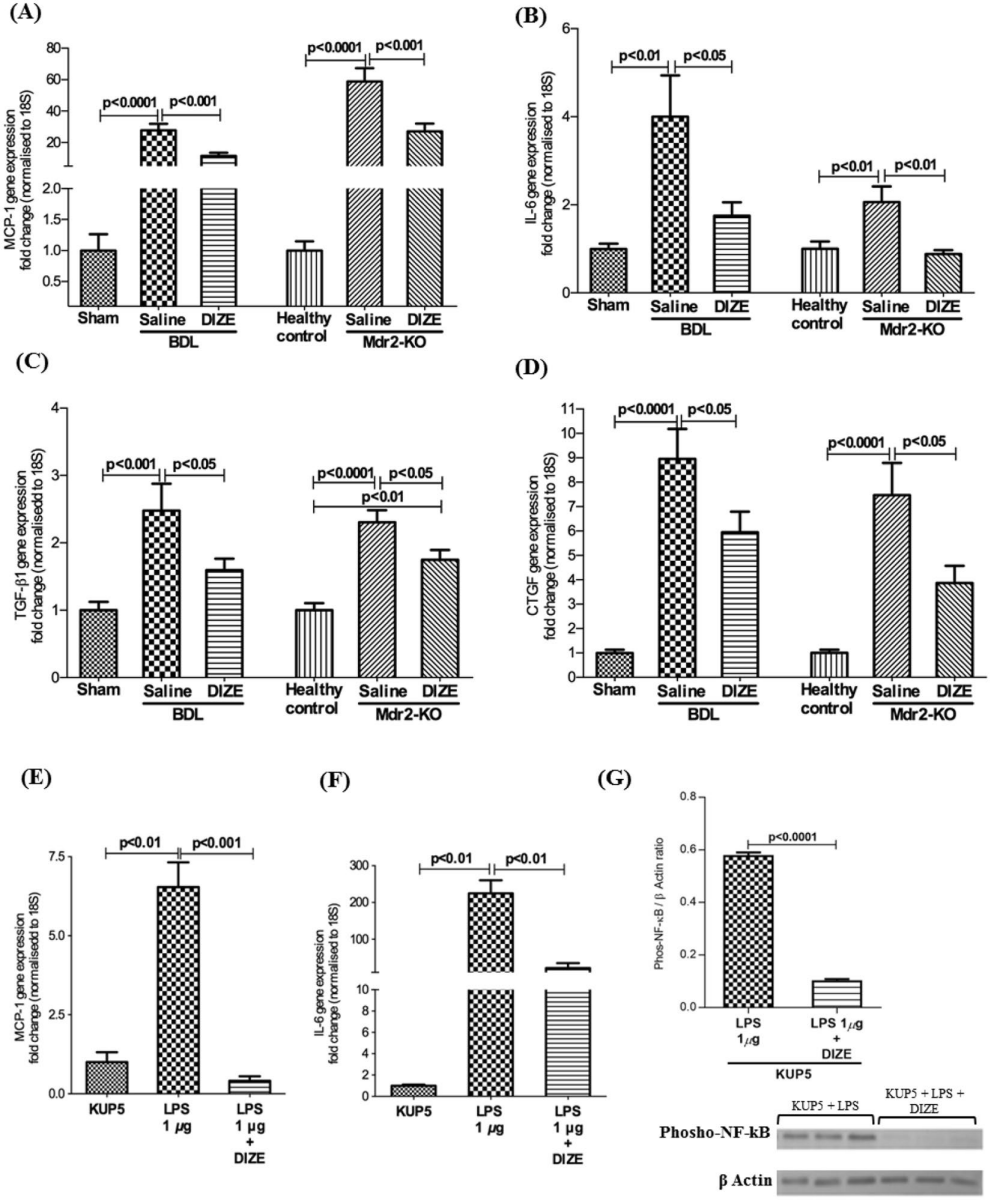
DIZE reduced proinflammatory and profibrotic cytokine gene expression. DIZE treated animals had a significantly reduced expression levels of hepatic proinflammatory genes, monocyte chemoattractant protein-1 (MCP-1) (**A**) and interleukin-6 (IL-6) (**B**) and profibrotic cytokine genes, transforming growth factor-β1 (TGF-β1) (**C**) and connective tissue growth factor (CTGF) (**D**) in both BDL and Mdr2-KO mice compared to those in saline-treated BDL and Mdr2-KO mice. Treatment with DIZE inhibited gene expression of MCP-1 (**E**) and IL-6 (**F**) and NF-kB activity (**G**) in lipopolysaccharide (LPS)-stimulated Kupffer cells (KUP5 cells). Each bar represents the mean ± SEM profile from 10–15 mice (panels A–D) or 3 independent experiments (panels E–G) per treatment group.

In biliary fibrosis, the activation of HSCs into myofibroblasts and the resulting increase in secretion of extra cellular matrix (ECM) is triggered by proinflammatory and profibrogenic mediators produced by activated resident macrophages, Kupffer cells and dying hepatocytes. Therefore, we investigated the effects of DIZE on gene expression of proinflammatory cytokines such as monocyte chemoattractant protein-1 (MCP-1) and interleukin-6 (IL-6) and profibrotic cytokines such as TGF-β1 and connective tissue growth factor (CTGF). As expected, the expression levels of these cytokines were up-regulated in BDL and Mdr2-KO mice compared with respective healthy controls (Fig. 3). Consistent with its antifibrotic effects, DIZE significantly down-regulated the expression of MCP-1 (Fig. 3A), IL-6 (Fig. 3B), TGF-β1 (Fig. 3C), and CTGF (Fig. 3D) in BDL and Mdr2-KO mice compared with those receiving saline infusion. Because Kupffer cells secrete proinflammatory cytokines, we investigated the expression of MCP-1 and IL-6 in KUP5 cells. We found that LPS caused an increased expression of MCP-1 (Fig. 3E) and IL-6 (Fig. 3F) in these cells. However, DIZE profoundly abrogated gene expression of these cytokines by KUP5 cells (Fig. 3E,F). The inhibitory effect of DIZE on cytokine secretion may be mediated by reduced NF-kB activity as the levels of phosphorylated NF-kB were significantly (p < 0.0001) downregulated by DIZE treatment in KUP5 cells (Fig. 3G).

### DIZE has no effect on hepatic ACE2 gene expression, its activity or intrahepatic angiotensin peptide levels

**Figure 4:**
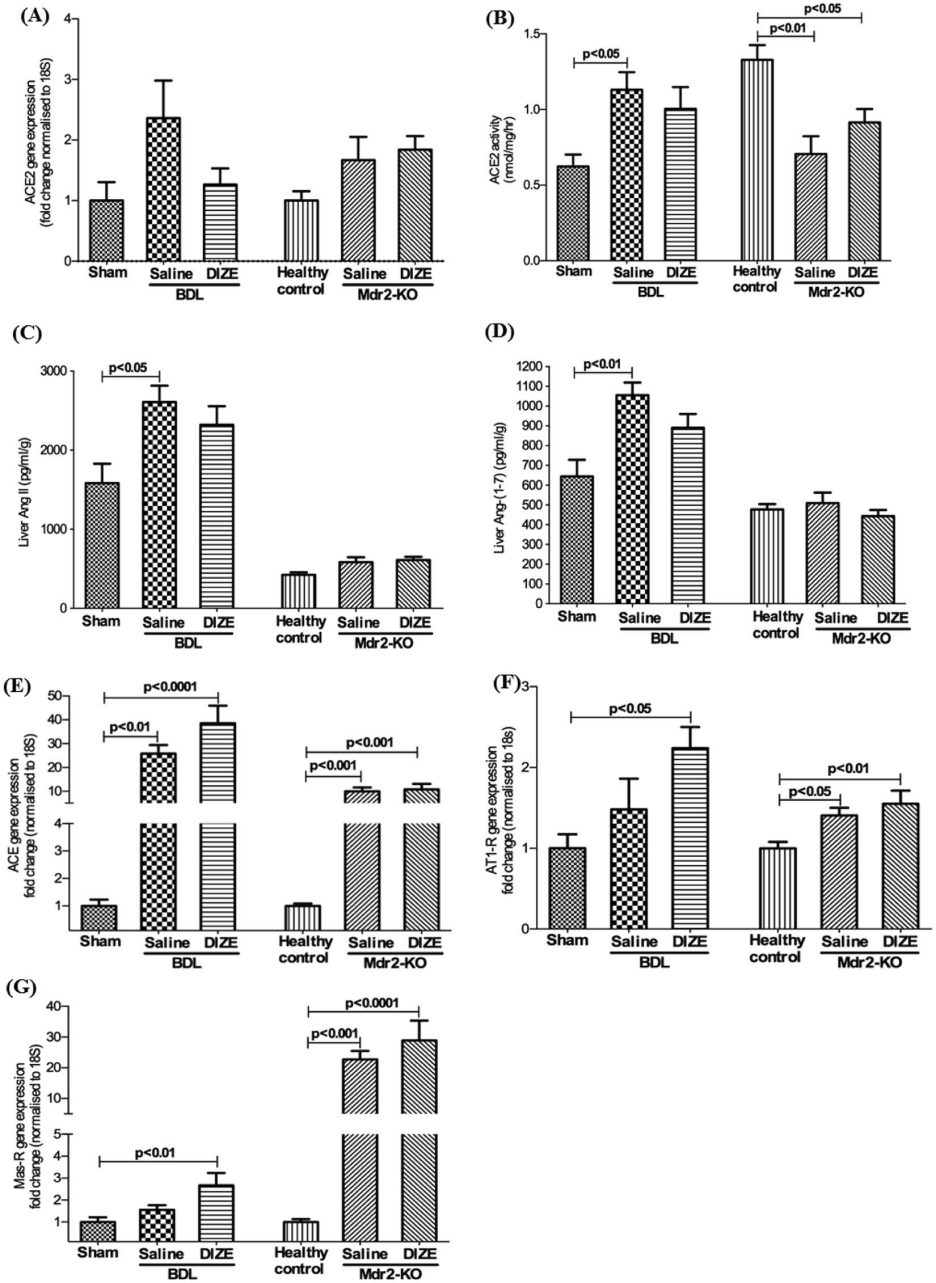
Diminazene aceturate (DIZE) had no effect on angiotensin converting enzyme 2 (ACE2) activity and on other RAS components. DIZE did not change the expression of ACE2 gene (**A**) or ACE2 protein activity (**B**) in BDL or Mdr2-KO mice. This was further confirmed by hepatic levels of angiotensin II (Ang II) (**C**) and angiotensin-(1-7) (Ang-(1-7)) (**D**) peptides which were not different between DIZE and saline treated BDL or Mdr2-KO mice. DIZE treatment in BDL and Mdr2-KO mice failed to affect gene expression of the RAS components such as angiotensin converting enzyme (ACE) (**E**), angiotensin II type 1 receptor (AT1-R) (**F**) and Mas receptor (Mas-R) (**G**) in the liver. Each bar represents the mean ± SEM profile from 10–15 mice per treatment group.

Some previous studies showed DIZE produces beneficial effects in a number of tissues by increasing ACE2 expression and activity^5,9,10^. We therefore assessed gene expression and activity of ACE2 and other RAS components in DIZE treated animals. In contrast to several published findings in other organs, we found that neither ACE2 expression and activity (Fig. 4A,B) nor Ang II (Fig. 4C) and Ang-(1-7) (Fig. 4D) peptide levels were affected by DIZE in BDL and Mdr2-KO mice. Furthermore, DIZE had no effect on other RAS components such as angiotensin converting enzyme (ACE), angiotensin II type I receptor (AT1-R) and Mas receptor (Mas-R) (Fig. 4E–G).

### DIZE inhibits gene expression and protein translocation of NOX subunits and MAPK signalling

Reactive oxygen species (ROS) generated by nicotinamide adenine dinucleotide phosphate oxidase (NADPH oxidase or NOX) activity function as key secondary messengers in numerous downstream signalling pathways in hepatic inflammation and fibrosis^15^. NOX signal transduces Ang II effects in hepatic stellate cells and is critical in hepatic fibrosis^16,17^. The NOX enzyme consists of several subunits and the activation of the enzyme is regulated by those subunits which are located in both the cell membrane and the cytosol of parenchymal (hepatocytes) and non-parenchymal (Kupffer cells and HSCs) cells. Therefore, we assessed gene expression levels of cell membrane-bound catalytic subunits NOX1, gp91^phox^/NOX2, and cytosolic regulatory subunit p47^phox^, a rate limiting subunit of NOX assembly which must be translocated into the cell membrane for enzyme activity^18^.

**Figure 5:**
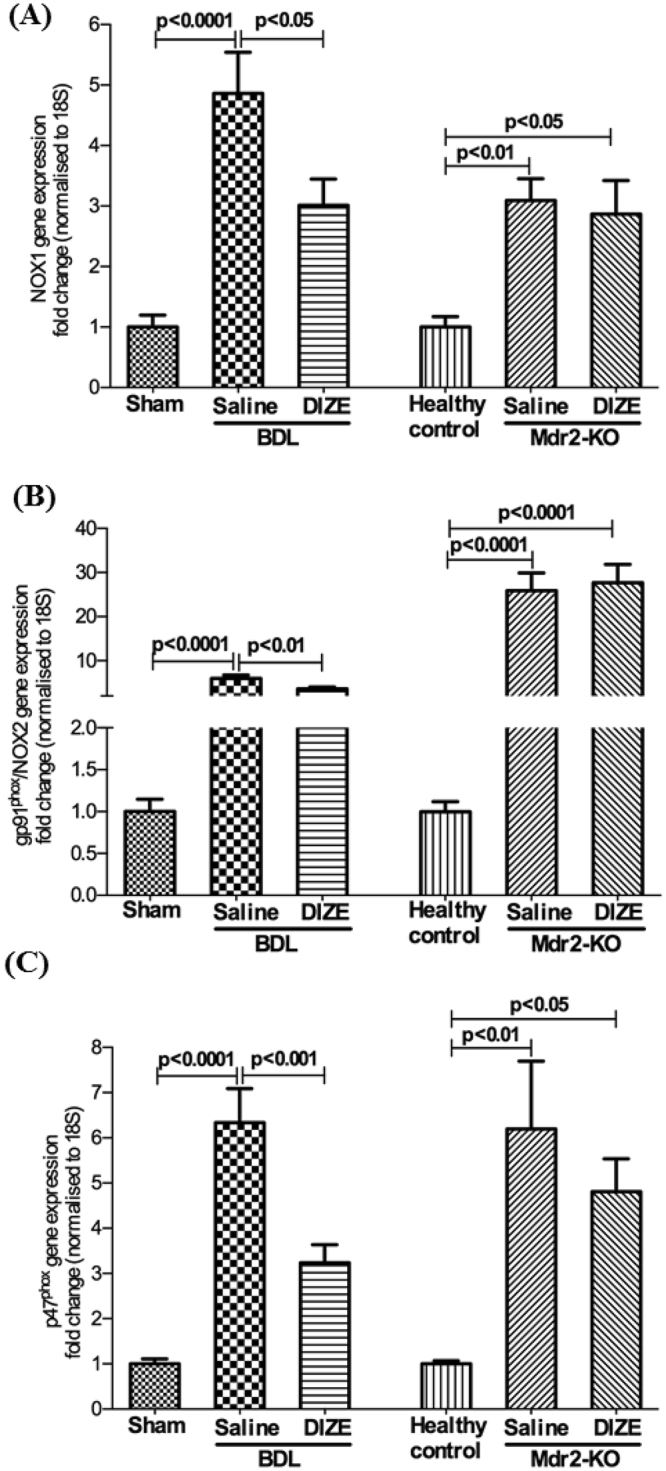
Diminazene aceturate (DIZE) inhibited NADPH oxidase (NOX) subunit gene expression. NADPH oxidase catalytic subunits, NOX1 (**A**) gp91^phox^/NOX2 (**B**) and regulatory subunit p47^phox^ (**C**) gene expressions were significantly up-regulated by bile duct ligation (BDL) or during disease progression in Mdr2-KO mice. However, DIZE inhibited the expression of these subunits in BDL but not in Mdr2-KO mice. Each bar represents the mean ± SEM profile from 10–15 mice per treatment group.

BDL surgery increased gene expression of the subunits compared to that in the sham-operated control mice. However, in response to DIZE treatment the expression of all three subunits of NOX were down-regulated in BDL mice compared to respective controls receiving saline infusion (Fig. 5A–C). In contrast, although the expression levels were significantly (p < 0.01) up-regulated in the diseased liver of Mdr2-KO mice, DIZE had no effect on subunit gene expression in Mdr2-KO mice (Fig. 5A–C).

**Figure 6:**
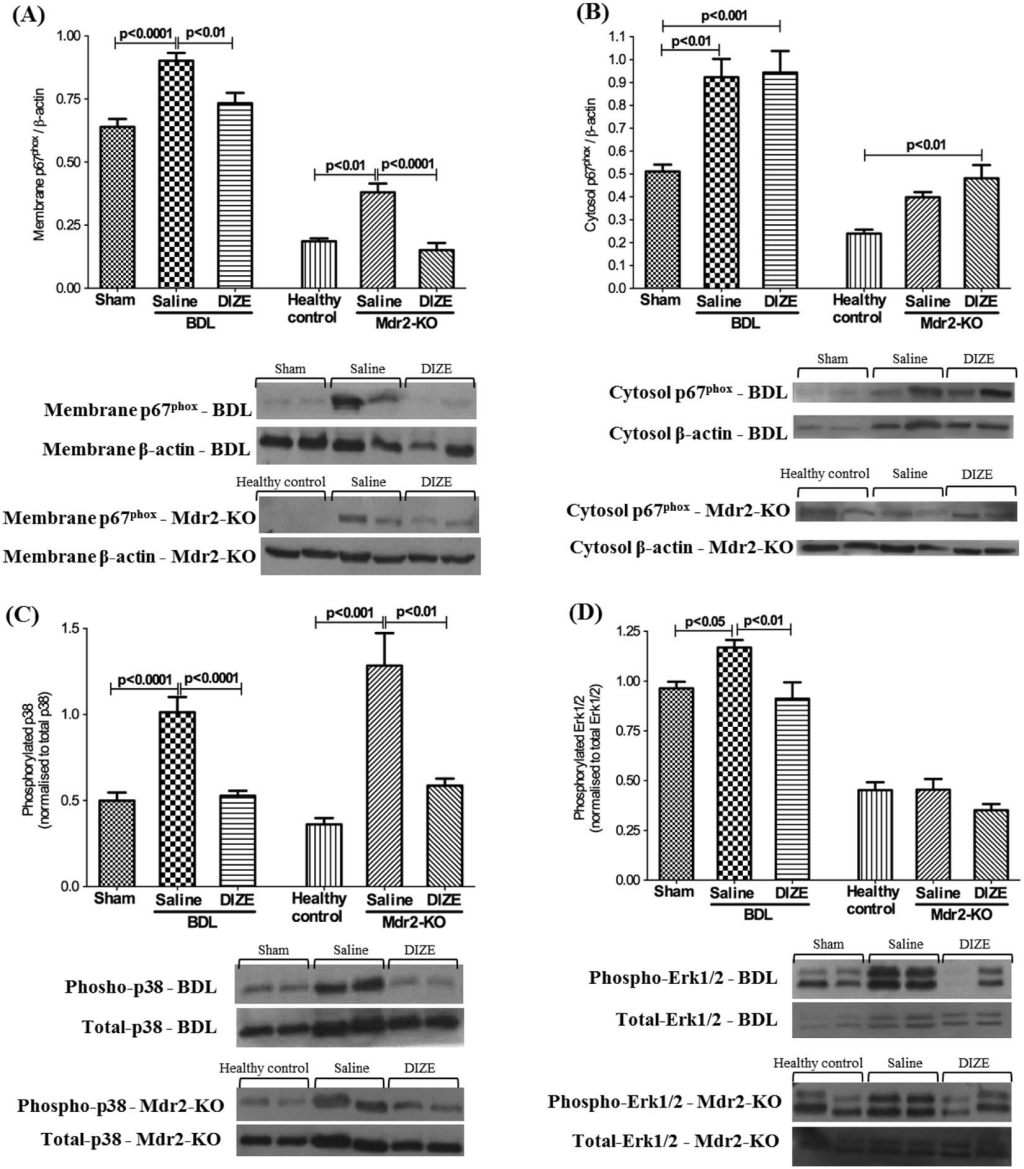
Diminazene aceturate (DIZE) inhibited NOX subunit protein translocation and activation of mitogen activated protein kinases (MAPK). Membrane translocation of regulatory subunit protein of NOX system, p67^phox^, was significantly increased in BDL and Mdr2-KO mice. (**A**) However, DIZE prevented the translocation of this rate-limiting subunit from the cytosol to the membrane in both BDL and Mdr2-KO mice. (**A**) On the other hand, DIZE had no effect on the protein levels in the cytosol (**B**) suggesting that it was subunit protein translocation that is affected by DIZE. The increased activity of MAPK, p38 (**C**) and Erk1/2 (**D**) which are redox-sensitive transcription factors, was inhibited by DIZE treatment. Each bar represents the mean ± SEM profile from 10–15 mice per treatment group.

Since translocation of NOX subunit proteins from the cytosol to the cell membrane is crucial for NOX activation which leads to ROS generation, we measured translocation of the rate-limiting subunit p67^phox^. We found that cell membrane (Fig. 6A) and cytosolic (Fig. 6B) protein levels of p67^phox^ subunit were increased in both animal models compared to respective healthy controls. However, in response to DIZE treatment, p67^phox^ protein level in the membrane was significantly reduced in both models compared to saline-treated disease controls (Fig. 6A), whereas DIZE treatment had no effect on p67^phox^ protein level in the cytosol (Fig. 6B), suggesting membrane translocation of the subunit was inhibited by DIZE treatment.

Moreover, there are close links between oxidative stress and activation of mitogen activated protein kinases (MAPKs), including p38 and Erk1/2^19^. We hypothesized that since MAPKs are redox sensitive intracellular transcription factors that are likely to decrease their activity in response to reduced oxidative stress, DIZE should decrease MAPK phosphorylation. Therefore, we evaluated the phosphorylated status of these molecules. Western blot analysis showed that in both liver fibrosis models, liver disease significantly increased the phosphorylation of p38 (Fig. 6C) and Erk1/2 (Fig. 6D). However, in keeping with its effects on NOX activity, DIZE treatment reduced phosphorylated p38 in both models and Erk1/2 in the BDL model.

### DIZE inhibits ROS generation

**Figure 7:**
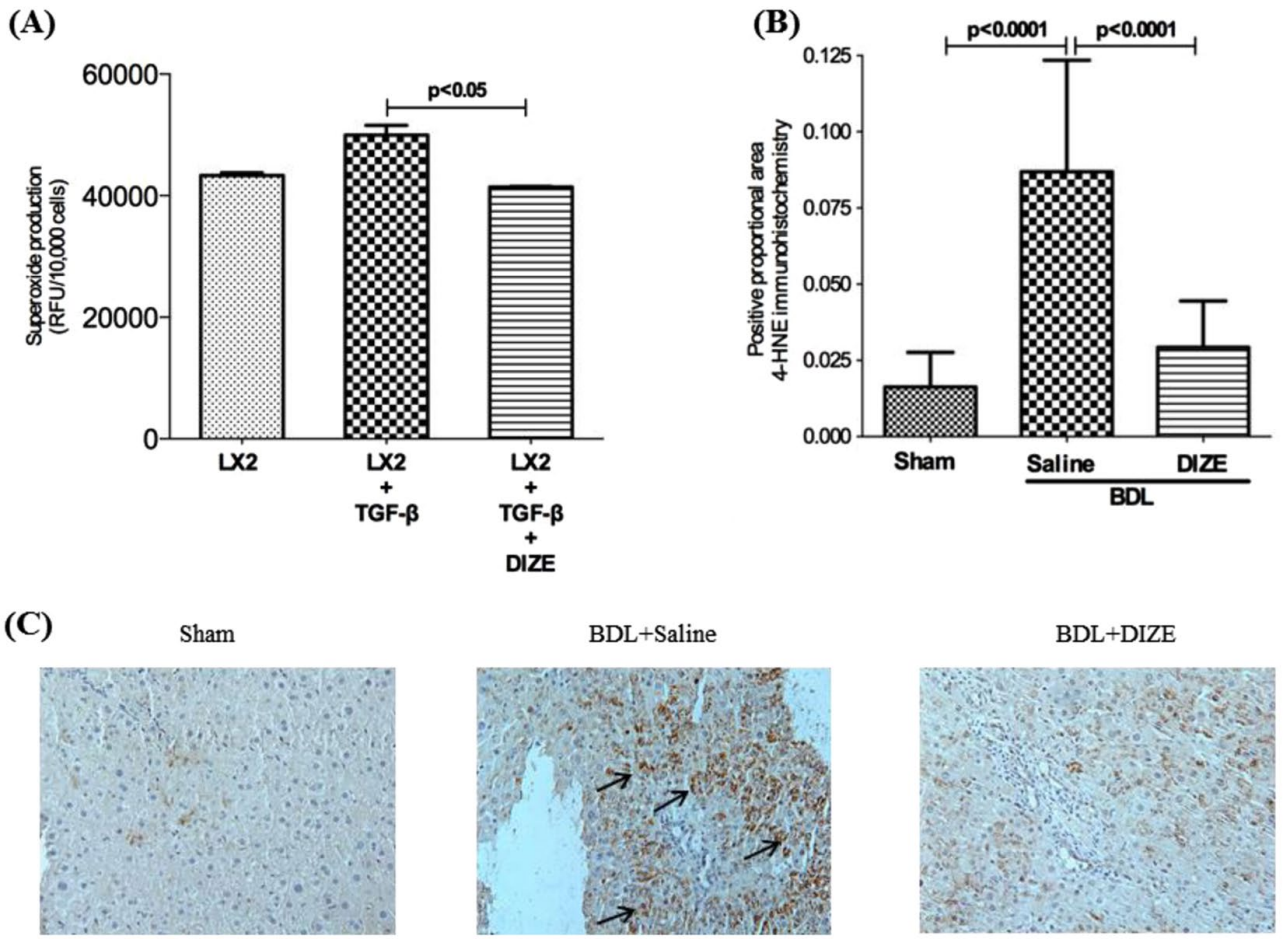
Diminazene aceturate (DIZE) inhibited reactive oxygen species (ROS) production and lipid peroxidation. Increased ROS production by TGF-β1 activated human hepatic stellate cells, LX-2 cells, was inhibited by incubation with DIZE. (**A**) ROS production by LX-2 cells was measured in a multiplate reader using 2′,7′-dichlorodihydrofluorescein diacetate. Increased oxidation of cell membrane lipids by ROS in the diseased liver was abrogated by DIZE, as measured by immunohistochemical localization of 4-hydroxynonenal (4-HNE) adducts. (**B**,**C**) Each bar represents the mean ± SEM profile from 3 experiments (**A**) or 10–15 mice per treatment group. (**B**) Representative images from the 3 groups are shown in panel C.

Consistent with its effects on membrane translocation of p67^phox^ subunit protein and MAPKs, including p38 and Erk1/2, we found that ROS production by TGF-β1 activated LX-2 cells was significantly inhibited by DIZE (Fig. 7A). Similarly, whilst bile duct ligation caused an increased (p < 0.0001) lipid peroxidation in the liver of BDL mice compared to sham-operation, there was a marked reduction (p < 0.0001) in lipid peroxidation in DIZE-treated mice compared to saline-treated control BDL mice (Fig. 7B,C).

### DIZE inhibits TGF-β1 mediated Smad2/3 pathway activation in human HSCs

**Figure 8:**
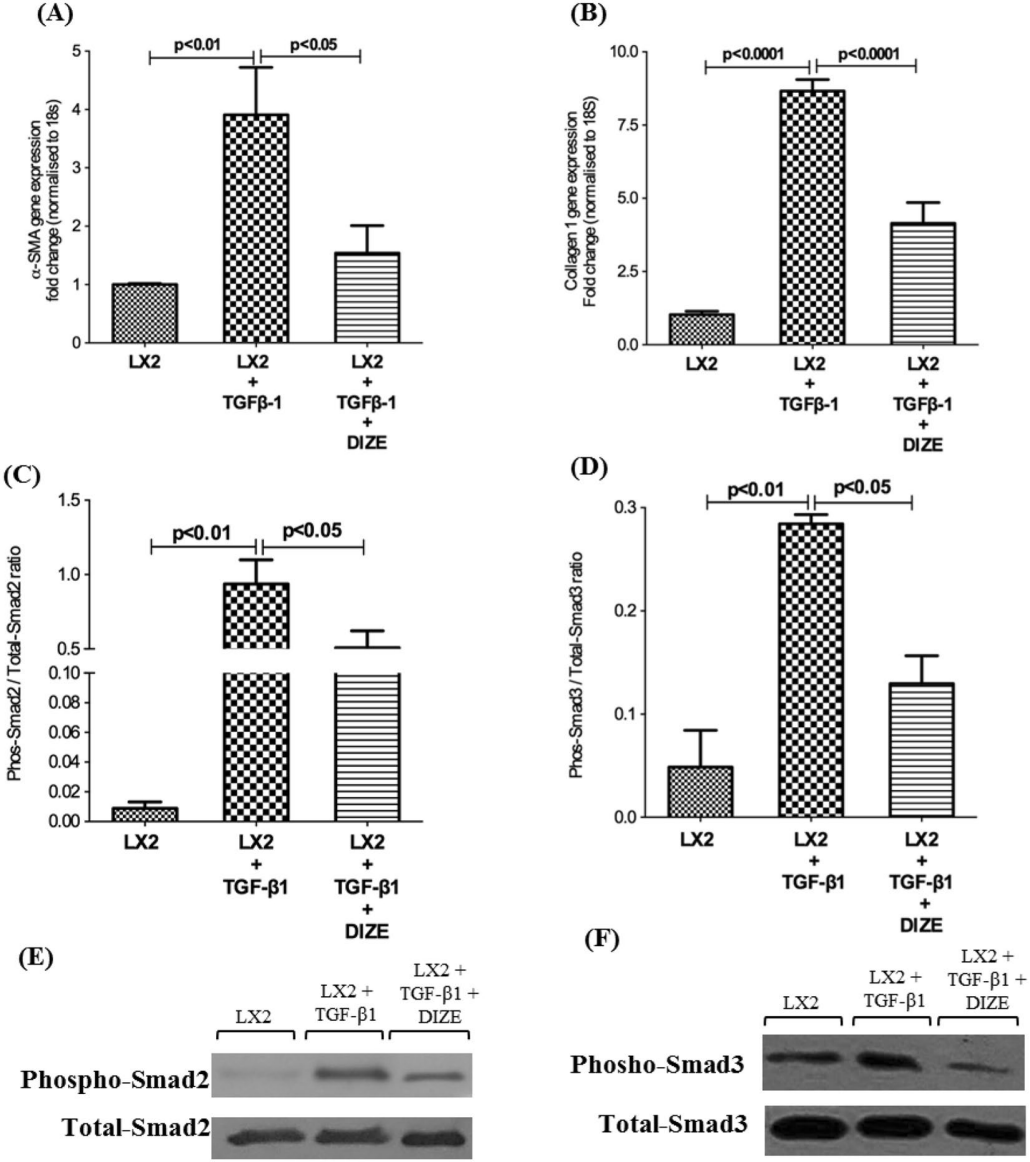
Diminazene aceturate (DIZE) inhibited human stellate cell activation and extracellular matrix secretion. TGF-β1 activated human hepatic stellate cells (LX-2 cells) showed a significantly increased gene expression of stellate cell activation marker, α-smooth muscle actin (α-SMA) (**A**), compared to untreated LX-2 cells. This was associated with increased expression of extracellular matrix gene, collagen 1 (**B**). However, DIZE treatment reduced both α-SMA and collagen 1 expression by approximately 50%. This reduction was accompanied by a significantly reduced phosphorylation of Smad2 (**C,E**) and Smad3 (**D,F**) proteins. Each bar represents the mean ± SEM profile from 3 independent experiments.

To further investigate the molecular mechanism(s) of DIZE action in biliary fibrosis, we studied the expression of the myofibroblast marker gene, α-SMA and ECM gene collagen 1 in activated human HSCs (LX-2 cells) after DIZE treatment. We found that increased activation of LX-2 cells, as reflected by increased expression of α-SMA (Fig. 8A) and ECM protein, collagen 1 (Fig. 8B) mediated by TGF-β1 was abrogated by DIZE treatment (Fig. 8A,B).

It has been well characterized that Smads are key intracellular signal transduction molecules activated by TGF-β1 in the liver^20^. We therefore investigated TGF-β1 downstream signalling by quantifying phosphorylated Smad2 and Smad3 proteins, the two most characterized intracellular signalling molecules of the classical TGF-β1 signalling pathway in liver fibrosis^20^. Western blot analysis of TGF-β1 induced LX-2 cells showed a marked increase of phosphorylated Smad2 (Fig. 8C) and Smad3 (Fig. 8D) compared to untreated LX-2 cells; however, the phosphorylation of these signal transduction molecules was reduced by treatment with DIZE.

## Discussion

In this study, we demonstrate for the first time that the small molecule drug, DIZE, markedly reduces biliary fibrosis in mice. DIZE had a therapeutic effect on established fibrosis in Mdr2-KO mice and also prevented the early development of biliary fibrosis in a short term BDL model. This antifibrotic effect of the drug was associated with a marked reduction in the release of proinflammatory cytokines, attenuation of cellular oxidative pathways, reduced profibrotic cytokine expression and inhibition of HSC activation.

The initial impetus for the current study was to further investigate the potential therapeutic role of ACE2 and the alternate axis of the RAS in biliary fibrosis, based on previous work showing that both ACE2 up-regulation and infusion of its peptide product Ang-(1-7) inhibited liver fibrosis in a range of animal models^21,22^. DIZE was chosen for this study as it is an FDA approved drug which has been reported to increase ACE2 activity. Indeed several studies suggest that, in other organs, DIZE elicits a range of beneficial therapeutic effects via this mechanism^5,9,10^. These effects of DIZE have been thought due to enhancement of ACE2 activity since the ACE2 inhibitor, C-16, was shown to block the therapeutic effects of the drug^9^. None of these previous studies, however, measured angiotensin peptide levels to determine whether the drug reduced tissue Ang II and increased tissue Ang-(1-7) levels, the mechanism thought to be primarily responsible for the beneficial effects of ACE2 activation.

In a marked contrast to these previous studies, we found that DIZE treatment did not affect ACE2 gene expression or activity. Furthermore, the hepatic levels of Ang II and Ang-(1-7) were not different between the disease controls and DIZE treated animals, strongly indicating that the effects of DIZE in the liver are ACE2 independent. Our results are consistent with those reported by Haber and colleagues who showed that the action of both DIZE or XNT another putative ACE2 activator, were independent of ACE2 activation^12^. This was established by showing that XNT was still able to lower blood pressure in ACE2 gene knockout mice and that both XNT and DIZE did not affect Ang II peptide degradation.

In chronic liver injury, fibrosis is driven by a range of proinflammatory cytokines such as TNF-α, MCP-1 and IL-6 released from activated Kupffer cells and HSCs^23^. These agents work in concert and stimulate each other in amplifying the fibrogenic response. For example, TNF-α, MCP-1 and IL-6 secreted by activated Kupffer cells provoke the activation of HSCs, whilst HSCs secrete MCP-1, which together with TNF-α further activates Kupffer cells^24,25^. Moreover, MCP-1 recruits inflammatory cells to the injured liver, thus perpetuating inflammation, HSC activation and subsequent fibrogenesis. We found that DIZE markedly inhibited the expression of not only hepatic TNF-α, MCP-1 and IL-6 but also their expression by activated Kupffer cells. A reduction in proinflammatory cytokine secretion by Kupffer cells is likely attributable to the inhibition of NF-kB activity by DIZE. Similar beneficial effects of the drug on the production of proinflammatory cytokines were documented in a model of pulmonary hypertension^5^. In the present study, in both models these changes were associated with reduced expression of the activated myofibroblastic marker, α-SMA, and of the profibrotic genes, TGF-β1 and CTGF and a subsequent reduction in collagen gene expression and ECM formation. Transforming growth factor-β1 is a critical regulator of liver fibrosis and a key profibrogenic stimulus in the activation of HSCs and subsequent secretion of ECM^26^. This potent cytokine works in concert with CTGF in the propagation of fibrosis^27^. Transforming growth factor-β1 activates downstream signalling pathways, in particular phosphorylation of Smad2/3 signal transducers with subsequent activation and transcription of TGF-β1 responsive genes, including fibrogenic gene expression^28–30^. We showed that TGF-β1 mediated activation of LX-2 cells and downstream phosphorylation of Smad2/3 proteins were down-regulated by DIZE, confirming that the intracellular signal transduction pathway of TGF-β1 in myofibroblastic cells is affected by DIZE treatment. Thus it is possible that DIZE reduced proinflammatory events, propagated by activated Kupffer cells in response to liver injury in BDL and Mdr2-KO mice, represents a mechanism by which the drug ameliorates HSC activation and subsequent inhibition of TGF-β1 signalling. However, we cannot rule out a possibility that DIZE may also have a direct effect on liver injury.

The generation of ROS via activation of NOX in HSCs is a key mechanism that propagates liver inflammation and profibrogenic responses, including the profibrotic effects of Ang II, and that this is dependent on translocation of different NOX subunits from the cytosol to the membrane^15,17^. We found that catalytic subunits NOX1 and NOX2 and the cytosolic regulatory subunit p47^phox^, the expression of which was increased in the liver of both models, was inhibited by DIZE in the BDL but not in Mdr2-KO mice. A lack of effect of DIZE on subunit gene expressions in the liver of Mdr2-KO mice is probably due to complex regulatory mechanisms documented for this model^31^. We then investigated the protein levels of another regulatory rate limiting NOX subunit, p67^phox^, and found that in line with gene expressions, cytosolic and membrane protein levels of p67^phox^ subunit were increased in both animal models. Unlike the effect on subunit gene expressions, in response to DIZE treatment, p67^phox^ protein levels in the membrane were markedly reduced in both models. This in turn, would be expected to reduce ROS generation. We showed that ROS production by activated human hepatic stellate cells, LX-2, was inhibited by DIZE. In support of this, we show that hepatic 4-HNE, a reliable biomarker of ROS-initiated lipid peroxidation^22,32^ was markedly reduced by DIZE treatment in the BDL model. These findings are consistent with other studies which showed that mice lacking the p47^phox^ protein subunit are protected from liver fibrosis induced by bile duct ligation^15^. Moreover, the NOX1 inhibitor, GKT-136901, has been shown to be able to suppress liver fibrosis induced by bile duct ligation^33^. Thus, the present study shows that whilst DIZE inhibits NOX subunit gene expression in the BDL model, DIZE-induced inhibition of membrane translocation of the regulatory p67^phox^ subunit in both models may be a key downstream mechanism by which DIZE reduces liver fibrosis. One important downstream consequence of ROS generation is the phosphorylation of MAPKs, including p38 and Erk1/2 which are redox sensitive potent transcription factors responsible for subsequent induction of proinflammatory and profibrotic cytokines^19^. We showed in the present study that DIZE treatment markedly reduced p38 and Erk1/2 phosphorylation in both biliary models, suggesting that by suppressing NOX activity, DIZE negatively regulates downstream redox sensitive signalling pathways. Collectively, these findings suggest that DIZE is a potent antifibrotic molecule that inhibits NOX activity with subsequent reduction in excessive ROS generation, oxidative stress and thereby improving biliary fibrosis.

It is known that necrosis and apoptosis of hepatocytes drive liver fibrosis in chronic liver injury. It has been shown that myofibroblast survival and perpetuation of ECM secretion is closely linked to phagocytosis of necrotic or apoptotic hepatocytes by myofibroblasts that is mediated via NOX, JAK/STAT, PI3K/Akt and NF-kB pathways^34,35^. In the present study, in BDL livers necrosis was markedly reduced by DIZE treatment, suggesting that the drug may also have a protective effect on hepatocytes. TNF-α, derived from Kupffer cells, is likely be an important trigger for hepatocyte necrosis in the BDL liver^36^. We showed that TNF-α expression in the liver and Kupffer cells as well as TNF-α protein secretion by activated Kupffer cells were reduced by more than 50% by DIZE treatment, suggesting that the inhibition of hepatocyte necrosis in DIZE treated animals may be mediated via this mechanism.

In conclusion, DIZE is a small molecule drug effective in ameliorating biliary fibrosis accompanied by reduced proinflammatory and profibrotic cytokine release, and inhibition of HSC and inhibition of ROS generation. We also demonstrate these effects are independent of ACE2 activation or changes in angiotensin peptide levels in the liver. The drug, which is commonly used in veterinary practice, is FDA listed and has been successfully used in clinical practice to treat human trypanosomiasis in tropical and subtropical countries. The present evidence suggests that DIZE has the potential to treat biliary fibrosis.

## Materials and Methods

### Animal models of liver disease

All animals were housed with a 12-hour light-dark cycle at room temperature (22 °C to 24 °C) with water and standard mouse chow *ad libitum*. Experimental procedures were approved by the Animal Ethics Committee of Austin Health and performed according to the National Health and Medical Research Council (NHMRC) of Australia Guidelines for animal experimentation.

### Short-term biliary fibrosis model – bile duct ligation (BDL)

Biliary fibrosis was induced in 6–8 weeks old, male C57BL/6 mice by double ligation and transection of the common bile duct as described previously^22^. The surgery was carried out under inhalation anaesthesia using 2% isoflurane (Piramal, USA) and oxygen mixture. At the time of bile duct ligation (BDL) surgery, two micro osmotic pumps (ALZET Micro osmotic pump, DURECT Corporation, Cupertino, CA) designed to deliver DIZE for 2 weeks were implanted subcutaneously into the supra scapular region of the mice (n = 12) at a dose of 10 mg/kg/day (each pump delivered 5 mg/kg/day). The saline infused BDL group (n = 11) was used as a treatment control to DIZE while the sham operated group (n = 12) was used as the healthy control group for the study. After 2 weeks of BDL, animals were euthanized using overdose of anaesthesia (pentobarbitone sodium-120 mg/kg) for blood and tissue collection. Micro osmotic pumps were collected at the time of sacrifice and the residual volumes were recorded to confirm the correct dose delivery of DIZE during the treatment period.

### Chronic biliary fibrosis model – Mdr2-KO mice

Mdr2-KO male mice at 14 weeks of age with FVB/N background were used in the study. These mice develop biliary fibrosis 3 weeks after birth and have established fibrosis which resemble human primary sclerosing cholangitis (PSC) at 3 months of age^37^. A group of Mdr2-KO mice received DIZE treatment (n = 14) from two subcutaneously implanted micro osmotic pumps designed to deliver the drug for four weeks at a dose of 10 mg/kg/day (each pump delivered 5 mg/kg/day). Mdr2-KO mice receiving saline (n = 15) was used as a study control to DIZE whereas FVB/N mice (n = 15) served as healthy control group for the study. Animals were euthanized after 4 weeks of treatment and samples were collected following the same procedure as for BDL model.

### Histological assessment of liver injury and fibrosis

Hematoxylin and eosin (H&E)-stained sections were assessed for inflammatory cell infiltration and parenchymal (hepatocyte) necrosis. Liver fibrosis was assessed by morphometric analysis of picrosirius red-stained area as previously described^22^. Refer to the supplementary material section for details.

### Liver biochemistry

Plasma ALT (alanine aminotransaminase), AST (aspartate transaminase) and ALP (alkaline phosphatase) were measured using a Beckman Coulter LX20 autoanalyser.

### Immunohistochemistry

Immunohistochemistry for α-smooth muscle actin (α-SMA) (1:50 dilution, Monoclonal 1A4, Dako Cytomation, Denmark) and 4-hydroxy-2-nonenal (4-HNE) (1:200 dilution, Alpha Diagnostic International, San Antonio, USA) were performed as described previously^22,32^. Refer to the supplementary material section for details.

### Quantitative real time polymerase chain reaction (qPCR) analysis

Total RNA was extracted from livers (50–100 mg) using TRIzol reagent (Life Technologies, Carlsbad, CA). qPCR was performed using multiplexing, as described previously^21,22^.

### Angiotensin converting enzyme 2 (ACE2) activity assay

Liver tissue ACE2 activity was measured as described previously^38^. Refer to the supplementary material section for details.

### Liver Angiotensin II and Angiotensin-(1-7) levels

Liver tissue (100–200 mg) was homogenized in 5 ml of 4 M guanidine thiocyanate with 1% trifluroacetic acid (Sigma Aldrich, St Louis, MO). The tissue homogenates were centrifuged at 50,000 × g for 20 minutes at 4 °C, supernatants were harvested immediately after the centrifugation and transferred into the pre-primed polymeric reversed phase strata cartridges (Phenomenex, Torrance, CA). The peptides were extracted from the tissue homogenate and eluted using elution buffer (Methanol:H_2_O:trifluroacetic acid in 80:19:1 ratio). Eluted angiotensin peptides were dried using speed vacuum centrifugation followed by resuspension in peptide assay buffer. Ang II and Ang-(1-7) concentrations were measured by radioimmunoassay (ProSearch International Australia P/I, Melbourne, Australia), as described previously^39^.

### Reactive oxygen species (ROS) measurement in LX-2 cells

LX-2 cells were cultured in clear bottom black 96-well plates (Corning Inc, Corning, NY) and were treated with transforming growth factor-β1 (TGF-β1) (10 ng/mL) (Sigma Aldrich,USA) for 24 hours, followed by 30 minutes incubation with 2′,7′-dichlorodihydrofluorescein diacetate (10 mM) (Sigma Aldrich) (see supplementary material for detailed protocol for LX-2 cell culture and maintenance). Cells were washed with 1 × PBS twice, replaced with new medium with the addition of 10 mM DIZE or PBS, and after 30 minutes of DIZE treatment the ROS generation from the cells was measured at 37 °C using a FLUOStar Optima plate reader (BMG LABTECH GmbH, Germany) with excitation and emission wavelength of 485 nm and 520 nm, respectively.

### Cell culture experiments

LX-2 cells (immortalized human hepatic stellate cells) were maintained and grown routinely as monolayer cultures in DMEM medium (Thermo Fisher Scientific, Waltham, MA), supplemented with 10% fetal bovine serum (FBS) (Sigma Aldrich), 4.5 g/l L-Glucose (Sigma Aldrich) and 1% penicillin and streptomycin (Thermo Fisher Scientific), with 5% CO_2_ in at 37 °C.

KUP5 cells (immortalized mouse Kupffer cells) were maintained and grown routinely as monolayer cultures in DMEM medium (Sigma Aldrich) supplemented with 10% fetal bovine serum (FBS) (Sigma Aldrich), 10 *μ*g/ml bovine insulin (Sigma Aldrich), 250 *μ*M monothioglycerol (Sigma Aldrich) and 4.5 g/l L-Glucose (Sigma Aldrich)^40^. The cells were treated with lipopolysaccharide (LPS) (1 *μ*g/ml) (Sigma Aldrich) for 24 hours, followed by either DIZE (0.1 mM) or saline for 24 hours. Total RNA isolated from LX-2 and KUP5 cells was used to determine gene expression by qPCR as described previously^21,22^. In addition, mouse TNF-α ELISA Kit (Abcam) was used to measure TNF-α protein level in KUP5 cell culture medium (100 *μ*l).

Both LX-2 and KUP5 cells were cultured in clear 6-well plates (Corning Inc, Corning, NY) and were grown into subconfluent state for experiments. Once the cells were adhered onto bottom of the plate, LX-2 and KUP5 cells were treated with transforming growth factor-β1 (TGF-β1) (10 ng/ml) (Sigma Aldrich) and lipopolysaccharide (LPS) (1 *μ*g/ml) (Sigma Aldrich), respectively, for 24 hours, followed by either DIZE (0.1 mM) or saline for 24 hours.

LX-2 and KUP5 cells were then harvested and homogenized in approximately 0.5 ml of ice-cold RIPA buffer supplemented with 1 × COMPLETE Protease Inhibitor Cocktail Tablet (Hoffmann-La Roche, Basel, Switzerland) and 1 × PhosSTOP Phosphatase Inhibitor Cocktail Tablets solution (Hoffmann-La Roche), then centrifuged at 16000 × g for 1 hour at 4 °C. After centrifugation, the resultant supernatant was kept and the protein was quantified using Pierce BCA Protein Assay Kit (Thermo Fisher Scientific).

### Western blotting

#### LX-2 cells and KUP5 cells

Monoclonal rabbit phospho-Smad2 antibody (Cell Signaling, Boston, MA), monoclonal rabbit phospho-Smad3 antibody (Cell Signaling) and monoclonal rabbit phospho-NF-kB (Cell Signaling) were used (overnight at 4 °C) for LX-2 and KUP5 cells. Thereafter, polyvinylidene fluoride (PVDF) membranes were incubated with polyclonal goat anti-rabbit HRP secondary antibody (Agilent Technologies, Santa Clara, CA) at room temperature for 1 hour. Monoclonal rabbit total Smad2 and Smad3 antibodies (Cell Signaling) for phosphorylated Smads, and β actin (Cell Signaling) for phosphorylated NF-kB were used as loading controls.

#### Preparation of membrane and cytosolic fractions of mouse liver

Liver tissue samples were homogenized in approximately 0.5 ml of ice-cold lysis buffer and 1 × PhosSTOP Phosphatase Inhibitor Cocktail Tablets solution (Hoffmann-La Roche) and then centrifuged at 72,000 × g using Optima TLX ultracentrifuge (Beckman Coulter) for 1 hour at 4 °C. After centrifugation, the resultant supernatant was kept as cytosolic p67^phox^ subunit samples, whereas the pellet was resuspended and sonicated in 0.5 ml ice-cold lysis buffer and kept as membrane p67^phox^ subunit samples. Liver protein samples (~50 *μ*g) from all mice were subjected to western blotting. Polyclonal rabbit p67^phox^ antibody (Sapphire Bioscience, Australia), monoclonal rabbit phosphorylated and total p38 antibody (Cell Signaling) and polyclonal rabbit phosphorylated and total extracellular signal-regulated kinase (Erk1/2) antibody (Cell Signaling) were used to probe liver protein extracted from BDL and Mdr2-KO mouse livers. Thereafter, PVDF membranes were incubated with polyclonal goat anti-rabbit HRP secondary antibody (Agilent Technologies). Total p38, total Erk1/2 were used as loading controls.

Blots probed with protein from LX-2 cells and the livers of BDL and Mdr2-KO mice were developed in enhanced chemiluminescence reagent (Thermo Fisher Scientific). Intensities of the digitally detected bands were evaluated densitometrically using Gel Doc XR System (BioRad, Hercules, CA).

### Statistical analysis

Means between groups were compared using either a two-tailed unpaired student’s *t*-test or one-way analysis of variance (ANOVA) with Tukey post-hoc test. All data are expressed as mean ± standard error of mean (SEM). All statistical analyses were carried out using the computer package PRISM (GraphPad Prism 7.0). p < 0.05 was considered statistically significant.

### Data availability statement

All data generated or analyzed during this study are included in this article.

## Electronic supplementary material

Supplementary Information
