# Supplementary material for: The small molecule drug diminazene aceturate inhibits liver injury and biliary fibrosis in mice
Source: Sci Rep. 2018 Jul 5;8:10175. doi: 10.1038/s41598-018-28490-y (PMC6033899; doi:10.1038/s41598-018-28490-y)
Supplement: Supplementary file 1 — Supplementary Information [file 41598_2018_28490_MOESM1_ESM.docx]

**The small molecule drug diminazene aceturate inhibits liver injury and biliary fibrosis in mice**

Indu G. Rajapaksha,^a,1^ Kai Y. Mak,^b,1^ Ping Huang,^c,^ ^1^ Louise M Burrell,^d,1^ Peter W. Angus,^e,2,^* Chandana B. Herath^f,1^

^1^Department of Medicine, The University of Melbourne, Austin Health, Heidelberg, Victoria, Australia.

^2^Department of Gastroenterology, Austin Health, Heidelberg, Victoria, Australia.

**Author email:**

^a^[indugrvet@gmail.com](mailto:indugrvet@gmail.com), ^b^[kai.mak@unimelb.edu.au](mailto:kai.mak@unimelb.edu.au), ^c^ling-yi.huang[@unimelb.edu.au](mailto:@unimelb.edu.au), ^d^[l.burrell@unimelb.edu.au](mailto:l.burrell@unimelb.edu.au), ^e^[peter.angus@austin.org.au](mailto:peter.angus@austin.org.au), ^f^[cherath@unimelb.edu.au](mailto:cherath@unimelb.edu.au)

**Key words:** Mdr2-knockout, bile duct ligation, ACE2, DIZE, antifibrotic

**Short title:** **DIZE inhibits biliary fibrosis**

***Correspondence should be addressed to:**

Professor Peter W Angus,

Department of Gastroenterology and Hepatology,

Austin Health, Heidelberg 3084, Victoria, Australia.

Tel + 61 3 9496 5582, Fax +61 3 9496 3487

Email: [peter.angus@austin.org.au](mailto:peter.angus@austin.org.au)

**Supplementary Information**

**Histological assessment of liver injury and fibrosis**

Four microns thick paraffin embedded liver tissue sections were mounted on silane-coated glass slides and stained with hematoxylin and eosin (H&E) and picrosirius red (BioScientific, Sydney, Australia) as described previously.^1^ H&E stained sections were assessed for inflammatory cell infiltration and parenchymal (hepatocyte) necrosis. Liver sections were assessed by a liver pathologist, and quantification of both liver injury and picrosirius staining was performed blinded to the animal groups. Collagen content of the liver was quantified using computerized image capture (MCID, Imaging Research, Ontario, Canada) for picrosirius red staining in the liver, as described previously.^2^ Picrosirius red staining was assessed in 10 fields/liver section at x200 magnification for both BDL and Mdr2-KO studies and the average value was used as one observation per animal.

**Immunohistochemistry**

Immunohistochemistry was performed on 4 *μ*m sections of paraffin embedded liver tissue sections mounted on silane-coated glass slides. Staining for α-smooth muscle actin (α-SMA), a marker of activated hepatic stellate cells (HSCs), and 4-hydroxy-2-nonenal (4-HNE), a marker of lipid peroxidation, was performed as described previously.^1,3^ Primary antibodies (α-SMA, 1:50 dilution, Monoclonal 1A4, Dako Cytomation, Denmark; 4-HNE, 1:200 dilution, Alpha Diagnostic International, San Antonio, TX, USA) were used with ARK Peroxidase (Dako, Agilent Technologies, Denmark) and StreptABComplex Duet Reagent Set (Dako, Agilent Technologies) respectively. The positive staining in each section (10 fields/liver section) was determined at x200 magnification using MCID and the average value was used as one observation per animal.

**Angiotensin converting enzyme 2 (ACE2) activity assay**

Liver tissue ACE2 activity was measured in homogenized (200 mg) liver samples by using specific quenched fluorogenic substrates (QFS, Auspep, Parkville, Australia) as described previously.^4^ The assay was performed with 50 *μ*M QFS in a final volume of 200 *μ*l of ACE2 assay buffer (100 mM Tris HCl, 1 M NaCl, pH 6.5). The fluorescence of the samples was measured at 37°C by using a FLUOstar Optima plate reader (BMG LABTECH GmbH, Ortenberg, Baden-Württemberg, Germany) as described previously.^4^

**References**

1 Mak, K. Y. *et al.* ACE2 Therapy Using Adeno-associated Viral Vector Inhibits Liver Fibrosis in Mice. *Mol Ther* **23**, 1434-1443, doi:10.1038/mt.2015.92 (2015).

2 Lubel, J. S. *et al.* Angiotensin-(1-7), an alternative metabolite of the renin-angiotensin system, is up-regulated in human liver disease and has antifibrotic activity in the bile-duct-ligated rat. *Clin Sci (Lond)* **117**, 375-386, doi:10.1042/CS20080647 (2009).

3 Goodwin, M. *et al.* Advanced glycation end products augment experimental hepatic fibrosis. *Journal of Gastroenterology and Hepatology* **28**, 369-376, doi:10.1111/jgh.12042 (2013).

4 Herath, C. B. *et al.* Portal pressure responses and angiotensin peptide production in rat liver are determined by relative activity of ACE and ACE2. *American Journal of Physiology - Gastrointestinal and Liver Physiology* **297**, G98-G106, doi:10.1152/ajpgi.00045.2009 (2009).
